# Ciliary Feature Counter: A program for the Quantitative Assessment of Cilia to Diagnose Primary Ciliary Dyskinesia

**DOI:** 10.3390/diagnostics10080524

**Published:** 2020-07-28

**Authors:** Andreia L. Pinto, Ranjit K. Rai, Claire Hogg, Thomas Burgoyne

**Affiliations:** 1 Paediatric Respiratory Medicine, Department of Paediatrics, Royal Brompton & Harefield NHS Trust, London SW3 6NP, UK; a.pinto@rbht.nhs.uk (A.L.P.); r.rai@rbht.nhs.uk (R.K.R.); c.hogg@rbht.nhs.uk (C.H.); 2 Department of Paediatrics, Imperial College London, London SW3 6LY, UK; 3 UCL Institute of Ophthalmology, University College London, London EC1V 9EL, UK

**Keywords:** cilia, primary ciliary dyskinesia, digital counter

## Abstract

Primary ciliary dyskinesia (PCD) is a disorder that affects motile cilia in the airway that are required for the removal of mucus, debris, and pathogens. It is important to diagnose PCD in early childhood to preserve lung function. The confirmation of a diagnosis relies on the assessment of ciliary ultrastructure by transmission electron microscopy (TEM). TEM involves the quantitative assessment of the ciliary ultrastructure to identify PCD defects as well as abnormalities resulting from infection. Many specialist diagnostic centres still rely on physical counters to tally results and paper notes to summarise findings before transferring the results to computer databases/records. To speed up the diagnostic data collection and increase the protection of patient information, we have developed digital ciliary feature counters that conform to the PCD reporting international consensus guideline. These counters can be used on a computer or tablet, and automatically generate notes regarding sample observations. We show that the digital counters are easy to use and can generate TEM diagnostic reports that will be useful for many PCD diagnostic centres.

## 1. Introduction

Primary Ciliary Dyskinesia (PCD) is a heterogeneous genetic disorder of the motile cilia. Cilia are hair-like structures which play an essential role in the airway by moving and clearing mucus, debris, and pathogens. PCD results in a failure to clear mucus from the airway, leading to chronic lung disease and rhinosinusitis, as well as impacting on hearing and fertility. It is estimated that the prevalence of PCD is approximately 1 per 10,000 births, but it is more common in populations where there is consanguinity [1]. A diagnosis of PCD at an early age is vital to implement appropriate treatment to preserve lung function and prevent lung damage. Both the European and North American diagnostic guidelines recommend that a diagnosis of PCD should be confirmed by transmission electron microscopy (TEM) in combination with another diagnostic test [2,3].

**Figure 1:**
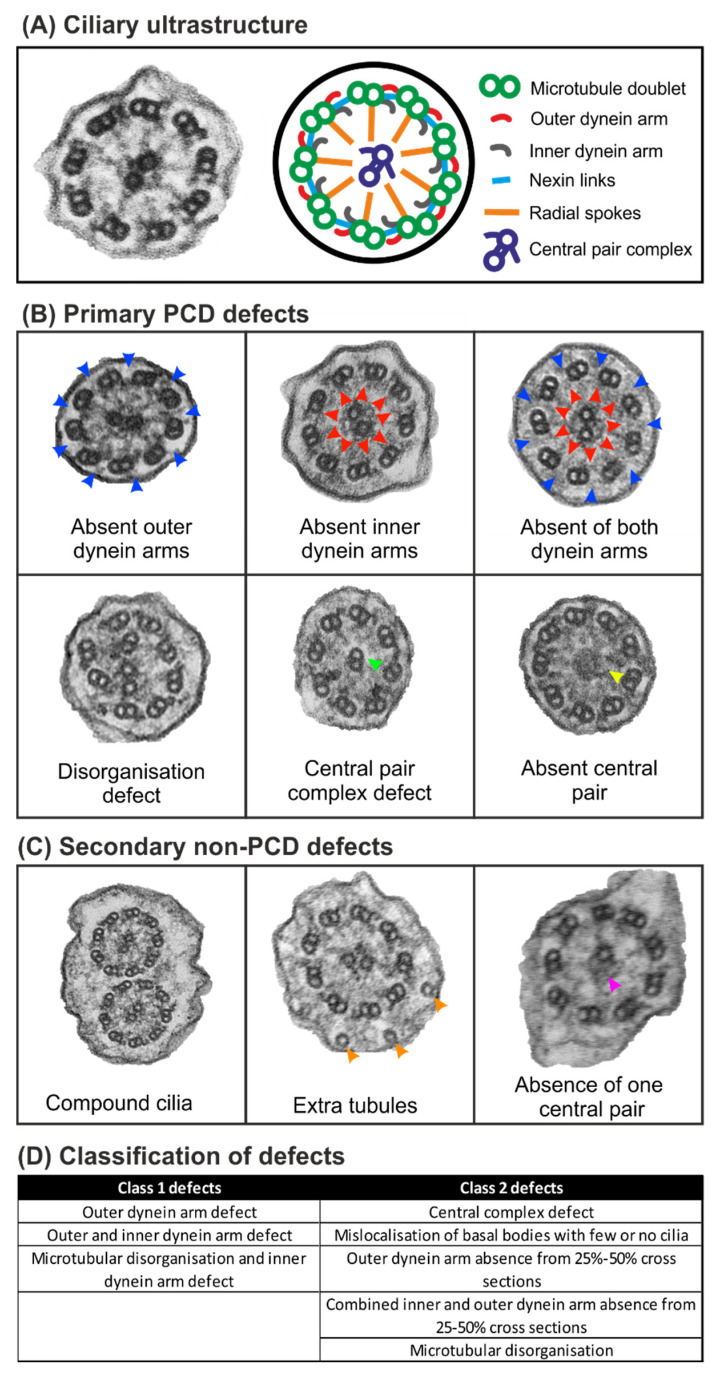
Normal healthy ciliary ultrastructure as well as common ciliary defects associated with primary ciliary dyskinesia (PCD) and secondary to infection. (**A**) Electron microscopy image and diagram of a cross section through a healthy respiratory epithelial cilium, highlighting the main ciliary components. (**B**) Common PCD defects, including the loss of structures or disarrangement of the cilia. The blue arrows indicate absent outer dynein arms, the red arrows indicate absent inner dynein arms, the green arrow indicates a microtubule doublet that has transposed into the centre of the cilium, and the yellow arrow indicates the absence of the central pair microtubules. (**C**) Some of the ciliary defects associated with unhealthy respiratory epithelium are caused by bacterial or viral infections. The orange arrow indicates an extra microtubule within the cilium, and the purple arrow indicates the loss of a single central pair microtubule. (**D**) The classification of primary PCD defects based on the international consensus guidelines for reporting TEM results [4].

PCD can present as a number of ultrastructural defects by TEM (Figure 1). These include the complete or partial absence of the outer, inner, or both dynein arms, as well as microtubular disorganisation or a central pair complex defect (Figure 1B). Due to infection, other secondary non-PCD defects are often present and include compound cilia, extra tubules, and the absence of one of the microtubules from the central pair complex (Figure 1C). As the dominant diagnostic assessment for PCD, there are international consensus guidelines for reporting TEM results which involve classifying PCD defects into two classes, as shown in Figure 1D [4].

**Figure 2:**
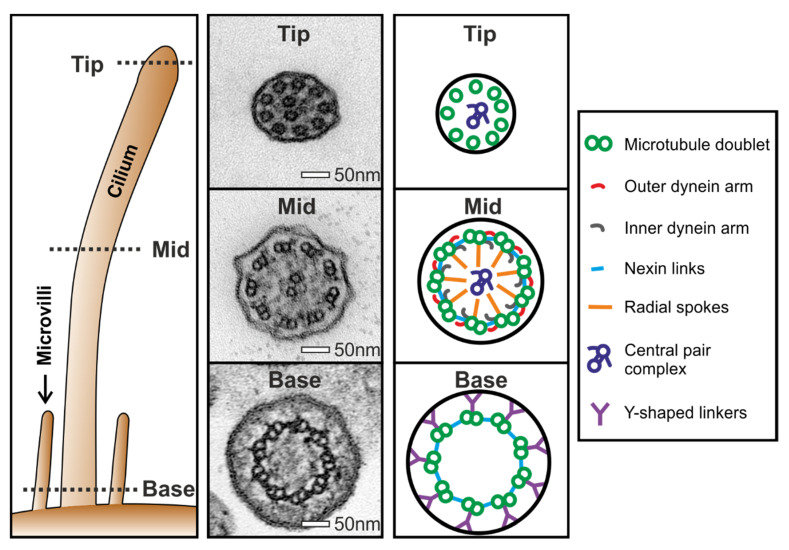
Ultrastructure of the different regions of a respiratory cilium. Respiratory cilia have a “9 + 2” microtubule arrangement (9 microtubule doublets and 2 single microtubules that make up the central pair), as can be seen in the mid region of the cilium. This ultrastructural arrangement runs throughout the cilium apart from at the tip and base where there are different ultrastructures, which include single microtubules and Y-shaped linkers, respectively, and an absence of dynein arms in both regions. When diagnosing PCD by TEM, it is vital to be aware of these differences in ultrastructure. In patients that have a central pair complex defect (see Figure 1B), typically the translocation of a microtubule doublet is observed at the tip, which makes it an important region of the cilium to examine.

**Figure 3:**
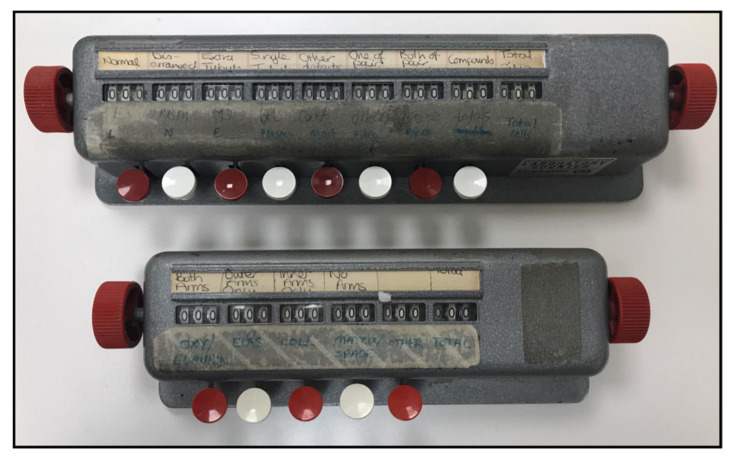
Physical counters used to count cilia features to assist in the diagnosis of PCD.

Fifty or more ciliary cross-sections need to be evaluated to reach a diagnosis for PCD, as outlined in the international consensus guidelines [4]. These need to be well-orientated cross-sections, and when assessing them it is vital to be aware of the ultrastructural differences at the base and tip compared to the rest of the cilium (see Figure 2). This involves tallying the assessments of the ultrastructure of each cilium as well as the dynein arms, which is often done using a physical counter (see Figure 3) and paper notes, to score patient cilia and make notes about each diagnostic patient sample. These notes are to be typed up onto a computer, which is time-consuming and poses a patient information confidentially risk. To speed up the collection of results directly to a computer system and to follow NHS (UK) and many international guidelines to phase out paper notes, we have developed a digital PCD ciliary counter. This easy-to-use counter can be used on a computer or tablet and works well with a touch screen, and it generates notes based on the simple selection of options that describe the diagnostic sample.

## 2. Materials and Methods

### 2.1. Preparation of Nasal Brushings for Ddiagnostic Electron Microscopy Aassessment

The ciliated respiratory epithelial cells were obtained using 3 mm bronchial cytology brushes inserted into subjects’ noses and brushing the nasal turbinate. The biopsies were placed into media 199 (Thermo Fisher Scientific, Waltham, MA, US) before fixing by adding an equal volume of 2.5% gluteraldehyde in 0.05 M sodium cacodylate buffer at pH 7.4 and left overnight or longer (up to 2 weeks) at 4 °C. The biopsies were incubated in 1% osmium tetroxide in 0.05 M sodium cacodylate buffer at pH 7.4 for 1 h and subsequently embedded in a 2% agar aqueous solution. The samples were dehydrated in increasing concentrations of methanol (70%, 90%, and 100%), followed by propylene oxide and mixtures of propylene oxide and araldite (1:1 and 1:3, respectively). Finally, the samples were incubated with araldite overnight to remove any propylene oxide and embedded in araldite at 65 °C for 72 hrs. Then, 90 nm-thick sections were cut from the araldite-embedded nasal brushings onto TEM grids and stained using 2% methanolic uranyl acetate for 7 min and Reynolds lead citrate solution for 5 min.

### 2.2. Diagnostic Electron Microscopy Assessment

Diagnostic sample sections were viewed on a TEM—in our case, a JEOL 1400+ TEM—and images were acquired using an AMT 16X CCD camera. Strips of ciliated epithelial cells were examined, and single cells were ignored (unless there were no sufficient strips of epithelia). For each strip of epithelia, the number of cells was counted and the ciliary ultrastructure was examined and quantified to determine if they were normal or had a defect (disarranged; extra tubule; single tubule; other defects, including the combined number of 8 + 2 (missing a microtubule doublet), 7 + 2 (missing two microtubule doublets) and 8 + 1 (transposed cilia, where the central pair is absent and a microtubule double is within the centre of the cilium) ciliary arrangements; missing tubule from central pair; missing central pair; or compound cilia) as well as the assessment of dynein arms (both arms present, inner dynein arm defect, outer dynein arm defect, or the absence of both arms). When the cilia were slightly oblique, the microscope stage was tilted to provide better orientated ciliary cross-sections. While examining the samples, additional notes were taken to determine the following: if the cilia have a normal orientation and longitudinal profile; the number of cilia that have an 8 + 2, 7 + 2 and 8 + 1 ciliary arrangement; as well as the relative amount of blood, ciliated strips, mucus, single cells, amount of cilia, inflammatory cells, and bacteria. Based on the counts and notes, a normal cilia or ciliary defect option was chosen from one of the following: normal; normal\unhealthy when there are secondary defects associated with infection and the presence of inflammatory cells and bacteria; class 1 defect (see Figure 1); class 2 defect (see Figure 1); or inconclusive due to unclear result or a small count. The counting and reporting follow the methods outlined in the international consensus guideline for reporting TEM results in the diagnosis of Primary Ciliary Dyskinesia [4].

### 2.3. Software Development

Both 32- and 64-bit versions of a basic and advanced counter to quantify ciliary features which assist in the diagnosis of PCD were designed using MATLAB 2020a and 2015b (Mathworks). The programs were built using the MATLAB GUIDE environment, allowing the generation of a graphical user interface and the programming of the underlying code. The two programs have been designed to be used while examining samples on the TEM, and they can be downloaded from the Figshare public repository (DOI —10.5522/04/12584675).

**Figure 4:**
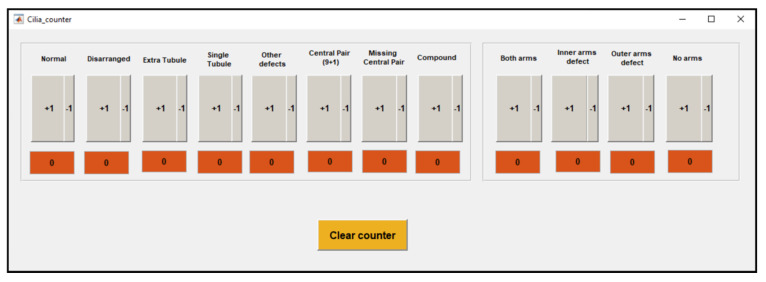
Basic digital counter to count cilia features to assist in the diagnosis of PCD.

The basic counter is a counter (Figure 4) to tally up ciliary features associated with PCD and secondary infection defects (see Figure 1 for the different types of defects). This has been designed to collect counts to subsequently input into the advanced counter. This approach may be popular with some people due to the large buttons in the basic counter. The counter has been programmed to tally (add one) when pressing the +1 button and to remove a value when pressing the −1 button. Values can also be added by typing numbers directly into the counter interface.

**Figure 5:**
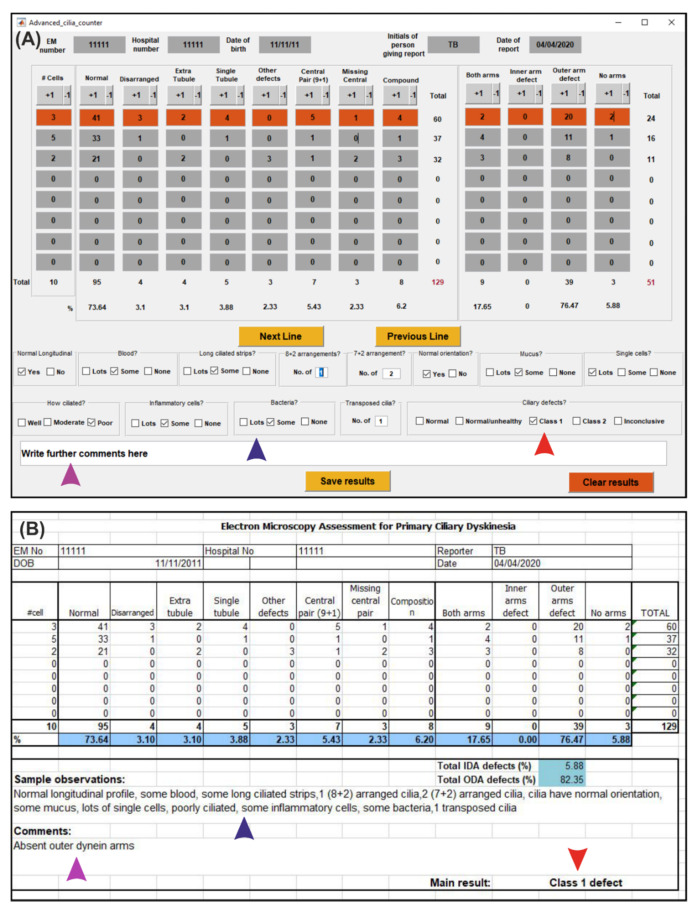
Advance counter that includes samples notes and an example of an Excel sheet that is generated when saving the results. (**A**) The number of features is tallied up by inputting numbers or by pressing the +1 or −1 buttons. The description of key features is inputted by pressing the checkboxes. (**B**) Excel sheet generated by the counter, which includes the counts of each type of feature and a description of the sample. The information entered by pressing the checkboxes in the counter populates a description in the Excel sheet (blue arrowhead in (**A**,**B**)). The ciliary defect class and comments about the sample are automatically transferred from the counter to the Excel sheet (red and purple arrowheads in (**A**,**B**) respectively).

The advanced counter includes a counter to quantify cilia at different regions of the diagnostic sample, with options to input sample information and notes (Figure 5). Similar to the basic counter, numbers can be tallied up or down by a value of one by pressing the +1 or −1 buttons, or values can be typed directly into the interface. The advance counter calculates the percentage of normal and defective cilia by dividing by the total number of cilia examined. If the values are modified by pressing +1, −1, or by entering a value, the percentages shown in the program are automatically updated. When viewing the sample on the TEM, there are checkboxes that represent the descriptors that can be used to provide additional notes about the sample. By saving the results, a Microsoft Excel sheet is generated that consists of the quantification of ciliary features and the checkboxes in the counter (blue arrowhead in Figure 5A) automatically populate a description of the sample (blue arrowhead in Figure 5B). The type of ciliary defect when selected in the counter (red arrowhead in Figure 5A) provides the main result (red arrowhead in Figure 5B), and comments are transferred to the Excel sheet (purple arrow in Figure 5A,B).

## 3. Results and Discussion

The counters provide an easy and efficient method to count ciliary features and make notes to assist in the diagnosis of PCD. We tested the advance counter by simultaneously collecting paper records of the diagnostic cilia feature counts and sample description when assessing 48 patient cases (taken by three individuals). The paper notes were compared to the values and information generated in the Excel sheets by the advance counter, and all the information was found to match without any observed errors. The Excel sheet generated contains all the information required to upload to an electronic patient record (EPR) system and to advise the consultant or clinician overseeing the referred patient. The checkboxes within the advance counter automatically populates the description of the sample observations in the Excel sheet (blue arrowhead in Figure 5A), which promotes the full assessment of each diagnostic sample, removes the risk of spelling mistakes or typos, and leads to a standardised terminology for each sample. This makes the results easier to interpret by the clinicians and is highly beneficial when sharing results across different diagnostic centres for research, auditing, and teaching purposes. By using the advance counter, the same diagnostic methods are the same as the outline in the international diagnostic consensus guidelines [4]. The images are assessed in the same way, but the counter can help to improve the sensitivity and specificity by reducing errors in record taking and maintaining standardised terminologies in the reports. Currently, it is difficult to calculate the sensitivity and specificity of the TEM-based diagnosis of PCD, as previously there lacked agreement in the reporting methods for TEM, and the consensus guidelines to overcome this have only recently been implemented [4]. Therefore, in the future it will be possible to accurately determine the sensitivity and specificity, once a substantial number of cases have been assessed by TEM by multiple diagnostic centres following these guidelines.

It is important to consider the results from other PCD diagnostic tests in combination with the TEM analysis [2,3]. These include genetic testing, measurements of cilia beat frequency, patterns from high speed video microscopy, and the immunofluorescence labelling of ciliary proteins. The results from these tests often correlate well with TEM—for instance, static cilia viewed by high-speed video microscopy are frequently associated with the absence of the outer dynein arm by TEM (see Figure 1B), as found in patients that have pathogenic variants in the *DNAH5* gene [5]. A limitation of TEM diagnostic analysis is that not all PCD defects are detectable by ultrastructural analysis. Approximately 25–30% of genetic mutations [6,7,8], including pathogenic variants in the *DNAH11* gene, cause ciliary defects that cannot be diagnosed by TEM [9]. When a normal diagnosis is given by TEM, the other diagnostic tests need to be considered to rule out PCD. Currently, there are approximately a quarter of PCD patients that have no genetic cause yet established, and in these patients a ciliary function and imaging assessment are required [3]. TEM provides a valuable tool in validating genetic test results, as there is a clear link between ultrastructural defects and many of the affected genes, and this can act as a guide in advancing our knowledge of PCD-causing mutations.

## 4. Conclusions

We demonstrate a ciliary feature counting system that can be of great benefit to diagnostic centres testing for PCD using TEM. This system follows international diagnostic consensus guidelines [4], making it relevant to groups globally. Physical counters are costly and hard to come by, thus the digital counter we demonstrate here provides a cost-free solution. As computer technology becomes an ever-growing trend in the diagnosis of disease, the digital counter system removes the need for paper records, and in the future it may be possible to integrate it into a machine learning system for the fully automated diagnosis of PCD.
